# Codesigned STAIR OF KNOWLEDGE intervention to prevent pressure ulcers, malnutrition, poor oral health and falls in Swedish nursing homes: an external pilot cluster randomised controlled study

**DOI:** 10.1136/bmjopen-2026-118018

**Published:** 2026-06-25

**Authors:** Merita Neziraj, Christine Kumlien, Malin Axelsson

**Affiliations:** 1 Department of Care Science, Malmö University, Malmö, Sweden; 2 Department of Cardiothoracic and Vascular Surgery, Skåne University Hospital, Malmö, Sweden

**Keywords:** Nursing Homes, Nursing research, GERIATRIC MEDICINE, Aged, Randomized Controlled Trial

## Abstract

**Objectives:**

This external pilot study aimed to evaluate whether a definitive trial of the STAIR OF KNOWLEDGE intervention is warranted. The specific outcomes concerned the trial procedure, including the recruitment, randomisation and retention of nursing homes and the clinical outcomes required for a definitive trial.

**Design:**

The study is an external pilot cluster randomised controlled trial.

**Setting:**

Swedish nursing homes participated.

**Participants:**

The inclusion criteria for participation in the study were nursing homes working with in the Swedish national quality registry Senior Alert. Data from 309 older persons were extracted from the registry. Of these, 214 were women and 95 men, ages 65–102 years.

**Intervention:**

The intervention aims to support nursing staff in providing preventive care regarding pressure ulcers, malnutrition, poor oral health and falls to older persons in nursing homes.

**Outcome measures:**

Following the Medical Research Council and the Knowledge to Action frameworks and guided by predefined progression criteria, we conducted an external pilot study to determine whether to proceed with a definitive trial. The outcome measures concerned testing nursing homes’ recruitment and randomisation procedures, their retention and the selection of the most appropriate primary outcome measures.

**Results:**

We recruited eight nursing homes and randomised four to each study arm. All participating nursing homes completed the study, supporting its overall feasibility. The data collection and analysis for the intended clinical outcomes were also feasible. A total of 309 older persons were included in the study sample and were identified as being at risk of pressure ulcers, malnutrition, poor oral health and falls. Among these, actions were planned for 299 (96.8%). Of those for whom actions were planned, actions were implemented on 270 older persons (90.3%). After 12 months, a reduction in implemented actions was observed.

**Conclusion:**

Conducting the current pilot study was a critical step towards preparing for a future definitive trial. The predefined progression criteria were successfully met, demonstrating the feasibility of the specific objectives—including the recruitment, randomisation and retention of nursing homes—as well as the data collection and analysis of intended clinical outcomes. The results of this external pilot study support proceeding with a definitive trial.

**Trial registration number:**

NCT05308862.

**STRENGTHS AND LIMITATIONS OF THIS STUDY**

- A strength is that this is the first pilot study to evaluate the entire preventive care process targeting pressure ulcers, malnutrition, poor oral health and falls among older persons in nursing homes, as outlined in the quality registry Senior Alert.
- A strength is that outcome measures were evaluated using predetermined progression criteria, including recruitment, randomisation and retention of nursing homes, as well as collection and analysis of data for the intended clinical outcomes.
- Randomisation imbalance between nursing homes is a limitation; therefore, stratified randomisation by nursing home type and size is recommended to ensure a more balanced allocation in the definitive trial.

## Background

 In nursing homes, nurse aides, registered nurses and managers (hereafter referred to as ‘nursing staff’) play a critical role in preventing common risks among older persons, including pressure ulcers, malnutrition, poor oral health and falls. To support preventive care for these risks, the Swedish web-based quality registry Senior Alert was established as a key resource for nursing staff working with older persons.^1^ Senior Alert provides evidence-based knowledge for a structured, systematic and individualised preventive care process targeting these risks. This preventive care process encompasses risk assessments using available, validated instruments; analyses of the causes when risks are identified; and the planning, implementation and evaluation of care actions.^2^

Despite the comprehensive support provided by Senior Alert, a gap persists between evidence-based knowledge and practice in nursing homes.^3^,^5^ As a result, the risks for pressure ulcers, malnutrition, poor oral health and falls are highly prevalent in Sweden: approximately 28% of older persons in nursing homes were at risk for pressure ulcers, 56% for malnutrition, 34% for poor oral health and 75% for falls.^3^ In fact, around 90% of older persons in nursing homes had at least one of these risks, 30% had two, 21% had three and 11% had all four.^3^ Moreover, alarmingly, not all older persons identified as being at risk for these conditions received planned care actions.^5 6^ Furthermore, there is often a mismatch between identified risks and the care actions that are subsequently planned and implemented.^7^,^9^

Insufficient knowledge among nursing staff seems to be a major challenge,^4 6^ and given that over 100 000 older persons live in Swedish nursing homes at some point during the year,^10^ the evidence–practice gap highlights an urgent need for a targeted educational intervention to better translate evidence-based knowledge into routine practice. Although educational interventions in this area do exist, they typically address only one or some of the risks for pressure ulcers, malnutrition, poor oral health and falls.^11^,^14^ Furthermore, previous systematic reviews reveal that educational interventions aimed at increasing knowledge for preventing these health risks often fall short due to methodological challenges.^11^,^14^ Therefore, healthcare interventions in this area are complex and require rigorous development and evaluation.^15 16^

Following the latest Medical Research Council framework for complex interventions,^17^ we previously developed an educational intervention, the STAIR OF KNOWLEDGE, aiming to prevent the risks for pressure ulcers, malnutrition, poor oral health and falls among older persons. This intervention was codesigned with nursing home staff and key municipal stakeholders, guided by the Knowledge to Action framework,^18^ to ensure contextual relevance throughout the development. A comprehensive description of both the development process and the intervention itself is provided in Neziraj *et al.*^19^ Following the subsequent phase in the Medical Research Council framework for complex interventions, we conducted a mixed-methods feasibility study (submitted), which found the intervention’s delivery, fidelity, acceptability and appropriateness feasible with minor amendments needed for a better fit to the local context. Accordingly, the next essential step was to evaluate the trial procedure through an external pilot study to determine whether to proceed with a definitive trial.^20^

### Aim

The external pilot study aimed to evaluate whether a definitive trial of the STAIR OF KNOWLEDGE intervention is warranted. The specific outcome measures of the current study pertained to the trial procedure, including the recruitment, randomisation and retention of nursing homes and the clinical outcomes required for a definitive trial.

## Methods

### Study design

The current study was designed as an external pilot cluster randomised controlled trial. It serves as a crucial stand-alone evaluation to justify and inform the planning of a subsequent definitive trial.^20^ As pilot studies are not intended for hypothesis testing, formal sample size calculations are not required. External pilot studies should instead focus on descriptive analyses,^20^,^22^ for instance, testing nursing homes’ recruitment and randomisation procedures, their retention and the selection of the most appropriate primary outcome measures.^20^ Moreover, the decision on whether and how to continue to a definitive trial^23^ should be informed by predefined progression criteria.^24^ In this study, the predefined progression criteria served as guidelines rather than rigid thresholds. They are reported in table 1 to ensure transparency in the evaluation process. The criteria regarding recruitment and retention were established pragmatically for the purpose of conducting the current study. Furthermore, the study was reported in accordance with the Consolidated Standards of Reporting Trial statement for randomised pilot and feasibility studies.^25^

**Table 1:** Outcome measures based on the predefined progression criteria to determine whether to proceed with a definitive trial

Outcome measures	Proceed	Proceed with changes	Do not proceed
Recruitment Can at least eight nursing homes take part in the study?	Recruit≥8 nursing homes	Recruit≤7 nursing homes	Recruit<7 nursing homes
Randomisation Can nursing homes be successfully randomised?	Logistically feasible		Not logistically feasible
Retention Can at least six nursing homes be retained in the study until completion?	≥6 nursing homes retained	<6 nursing homes retained	<5 nursing homes retained
Intended clinical outcomes Can data on the clinical outcomes be collected and analysed as intended?	Feasible		Not feasible

Outcome measures based on the predefined progression criteria to determine whether to proceed with a definitive trial (green indicates proceed, amber indicates proceed with changes and red indicates do not proceed).

### Setting

Responsibility for Swedish nursing homes lies with the municipalities, which provide most health and medical care within their jurisdiction. In nursing homes, older persons receive continuous care primarily by nurse aides, who deliver the round-the-clock care and services in accordance with the Social Services Act. In addition to their caregiving duties, nurse aides are delegated specific nursing tasks by registered nurses under the Health and Medical Services Act. Nurse aides usually have a secondary degree in nursing, involving 3 years of study in high school, but they do not necessarily have to have any formal education in nursing. Registered nurses have a bachelor’s degree in nursing, which involves 3 years of study at university, and they retain overall responsibility for the care provided in nursing homes. Nurse aides are also supervised by managers, who are based within respective nursing homes, whereas registered nurses are supervised by managers located externally. Managers usually have a bachelor’s degree, but not necessarily in nursing.

### Intervention: development, content, format and delivery

The STAIR OF KNOWLEDGE intervention was codesigned with nursing staff following the Medical Research Council framework for complex interventions and the Knowledge to Action framework KTA framework^17 26^ to support them in accomplishing preventive care aligned with evidence-based practice. Accordingly, it consists of multiple components.^19^ The description of the intervention followed the template for intervention description and replication.^27^ A detailed description of the development and content of intervention has previously been provided in Neziraj *et al*,^19^ but in brief, the intervention includes a foundation module and six sequential stairs, each targeting key aspects of the preventive care process for pressure ulcers, malnutrition, poor oral health and falls. It comprises both reading and video content, offered through both individual and group sessions. Its format was purposefully designed to be structured, readable and visually appealing. The intervention was delivered over an 8-week period from September to November 2022. Each nursing home in the intervention arm received the intervention over a 3-week span, with overlapping schedules across the nursing homes. Meanwhile, the nursing homes in the control arm continued their standard practice of working according to Senior Alert without changes.

### Patient and public involvement

The project was carried out in close collaboration with nursing staff and other relevant stakeholders, such as the head within the municipality, managers in charge of nursing homes, head of managers in charge of registered nurses, development managers and head of unit for research and development and centre of competence. They were actively engaged throughout the development and evaluation phases of the intervention.

### Clinical outcomes: data extraction and statistical analysis

The inclusion criteria were nursing homes working with the Swedish national quality registry Senior Alert. Data were extracted from the registry Senior Alert covering the period from 12 March 2022 to 12 September 2023. The open cohort included all older persons registered in Senior Alert during the timeframe. This timeframe encompassed three points: 6 months before the intervention started, 0–6 months following the intervention and 6–12 months following the intervention. During this timeframe, data on risk assessments were extracted from the following instruments: the Modified Norton Scale^28^ for pressure ulcers, the short form of the Mini Nutritional Assessment^29^ for malnutrition, the Revised Oral Assessment Guide-Jönköping^1 30^ for poor oral health and the Downtown Fall Risk Index^31^ for falls. Additionally, during this timeframe, data on both planned and implemented actions for older persons identified as at risk were extracted from the registry. Data on biological sex and age were also extracted.

All analyses were performed using IBM SPSS Statistics for Windows, V.30.0 (IBM Corp, Armonk, New York). To describe the study sample, we applied the following descriptive analyses: percentage, frequencies, mean and SD.

## Results

The findings are reported based on the specific study outcomes.

### Recruitment of nursing homes

Nursing homes were eligible if they used the quality registry Senior Alert and were located in a municipality in southern Sweden. The recruitment process was carried out in several steps to ensure informed participation. Initially, a reference group comprising nurse aides, nursing home managers, head of managers for registered nurses and development managers was formed to establish and facilitate contact with key stakeholders in the municipality. The head of nursing homes in the municipality was then informed about the study and subsequently disseminated the information to the managers of all nursing homes in the municipality through internal meetings. Out of 39 (100%) nursing homes in the municipality, 5 (13%) did not meet the inclusion criteria, and 13 (33%) stated other reasons for not participating, such as other ongoing projects in the nursing home or a new manager in the nursing home.

**Figure 1:**
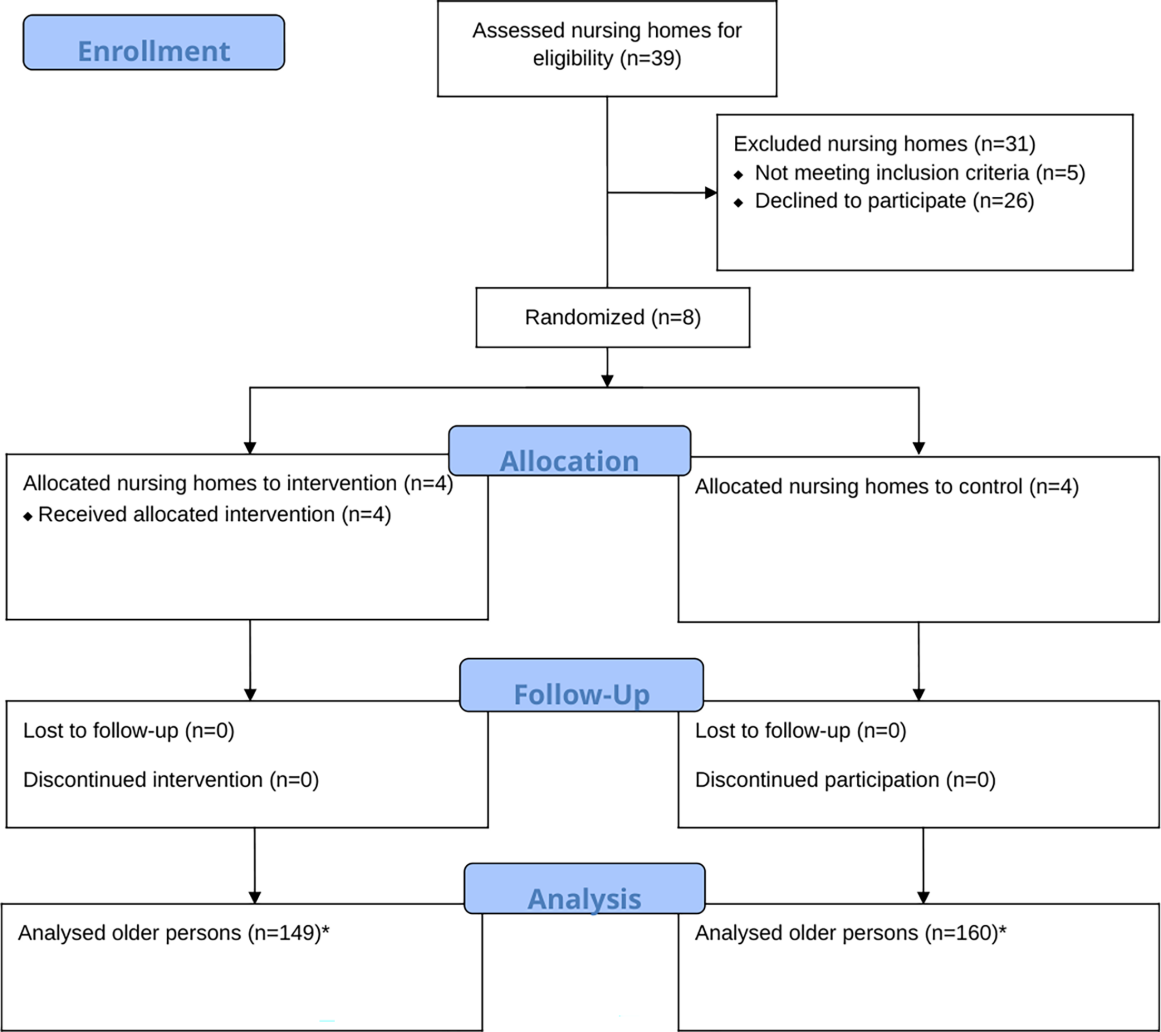
Flow diagram of the enrollment process. *Data for 59 older persons were missing, leading to their exclusion from the study. The final study population consisted of 309 older persons. CONSORT, Consolidated Standards of Reporting Trials.

Thereafter, the first author MN was invited to attend digital meetings with the managers of the eligible nursing homes (n=21) to present the study and answer any questions. The managers were then provided with a formal information letter outlining the study’s aim and procedures. In total, the managers of eight nursing homes (38%) agreed to participate (see figure 1 for the enrolment process). Thus, they received a prerecord video designed to be shared with nurse aides and registered nurses within their respective nursing homes, ensuring that they were adequately informed and engaged.

The managers were then given an opportunity to ask questions before they provided written informed consent and completed the baseline background characteristics of their nursing homes. Table 2 shows the baseline background characteristics of the nursing homes in the intervention and control arms, including the type of nursing homes, number of older persons living there and the nursing staff working there.

**Table 2:** Baseline background characteristics of nursing homes in the intervention arm (n=4) and the control arm (n=4)

	Type of nursing home	Older persons N	Nurse aides N	Registered nurses N	Managers N
Intervention arm					
Nursing home 1	Dementia care unit	31	33	2	1
Nursing home 2	Dementia care unit	24	25	1	1
Nursing home 3	Dementia care unit	32	35	2	1
Nursing home 4	Nursing home and dementia care unit	31	28	1	1
Total, N		118	121	6	4
Control arm					
Nursing home 1	Nursing home and dementia care unit	41	37	2	1
Nursing home 2	Dementia care unit	72	75	4	2
Nursing home 3	Nursing home	27	21	2	1
Nursing home 4	Nursing home and dementia care unit	44	41	2	2
Total, N		184	174	10	6
**Total, N**		302*	**295**	**16**	**10**

* This number reflects the older persons at this specific time. During the study period, older persons moved into the nursing homes (or died).

### Randomisation procedure

The eight participating nursing homes served as clusters and were the allocation units. They were randomised into either the intervention or control arm prior to the intervention. Using a formula in Microsoft Excel 365, the first author MN generated a randomisation list and subsequently informed the nursing home managers in both the intervention and control arms of the allocation output. However, as shown in table 2, the randomisation resulted in notable baseline imbalances between the intervention and control arms. These imbalances included differences in nursing home type, the number of older persons living there and the number of nursing staff working there.

### Retention of nursing homes

None of the eight nursing homes dropped out during the study period.

### Clinical outcomes

The following findings, pertaining to both the intervention and control arms, are presented in accordance with the preventive care process as outlined by Senior Alert.

#### Demographics

Older persons were eligible if they were registered in Senior Alert as undergoing the preventive care process for the risks of pressure ulcer, malnutrition, poor oral health and falls during the study period (ie, 6 months before the intervention started, 0–6 months after and 6–12 months after). Thus, of the 368 older persons registered in Senior Alert during the study period, 309 were registered as being at risk for pressure ulcer, malnutrition, poor oral health and falls and were therefore included in the final study sample. The study population (n=309) consisted of 214 women (69.3%) and 95 men (30.7%), and their ages ranged from 65 to 102 years, with a mean age of 86.8 years (SD 7.7). The mean age was 88.2 (SD 7.5) among women and 83.7 (SD 7.1) among men.

Among the older persons included in the study (n=309), 149 (48.2%) lived in nursing homes allocated to the intervention arm, and the remaining 160 (51.8%) were allocated to the control arm. The intervention arm comprised 94 women (63.1%) and 55 men (36.9%), whereas the control arm comprised 120 women (75.0%) and 40 men (25.0%). In the intervention arm (n=149), the age ranged from 64 to 102 years, with a mean of 84.68 years (SD 7.3). In the control arm (n=165), the age ranged from 64 to 101, with a mean of 87.33 years (SD 7.4).

#### Risk assessments

A total of 753 risk assessments were registered in Senior Alert across both intervention and control arms (n=309), of which 521 (69.2%) were registered for women and 232 (30.8%) for men. Each older person was assessed for the risk of pressure ulcers, malnutrition, poor oral health and falls at each assessment point. However, the number of risk assessments per older person varied throughout the study period, ranging from 1 to 6, with a mean of 2.44 risk assessments per older person (SD 1.3). Specifically, 92 (29.8%) older persons were risk assessed once, 88 (28.5%) twice, 69 (22.3%) three times, 27 (8.7%) four times and 28 (9.1%) five times; only five (1.6%) older persons underwent all six risk assessments. Among the older persons included in the study (n=309), 122 (39.5%) were identified as being at risk for pressure ulcers across all assessment points. A higher proportion, namely 221 (71.5%) older persons, was identified as being at risk for malnutrition across all assessment points. For poor oral health, 140 (45.3%) older persons were assessed as being at risk across the six assessment points. The highest proportion was observed for the risk for falls, with 266 (86.1%) older persons receiving this assessment across all assessment points.

Of the total 753 risk assessments registered throughout the study period, 393 assessments (52.2%) were registered in the intervention arm and 360 assessments (47.8%) in the control arm. The number of registered risk assessments varied between the different time points of the study period for the intervention and control arms. In the intervention arm, 148 (37.6%) risk assessments were registered 6 months before the intervention, 137 (34.9%) 0–6 months after the intervention and 108 (27.5%) 6–12 months after the intervention. In the control arm, 114 (31.7%) risk assessments were registered 6 months before the intervention, 155 (43.1%) 0–6 months after the intervention and 91 (25.3%) 6–12 after the intervention.

#### Planned actions

Actions were planned for 299 of the 309 older persons identified as at risk, representing 96.8% of the total sample. The 299 persons had a total of 705 planned actions during the study period: 334 actions (47.4%) in the intervention arm and 371 actions (52.6%) in the control arm. Specifically, in the intervention arm, 140 actions (41.9%) were planned 6 months before the intervention, 135 (40.4%) 0–6 months after the intervention and 96 (28.7%) 6–12 months after the intervention. In the control arm, 104 actions (28.0%) were planned 6 months before the intervention, 153 (41.2%) 0–6 months after the intervention and 77 (20.8%) 6–12 months after the intervention. However, as shown in table 3, the intervention arm involved more planned actions for some of the risks 0–6 months after the intervention, compared with the control arm. For detailed information on actions planned for different risks in both the intervention and control arms at different assessment points, see table 3. Table 3 also presents information on actions planned for older persons who were *not* identified as being at risk.

**Table 3:** Planned actions (n=705) for older persons assessed for the risks of pressure ulcers, malnutrition, poor oral health and falls in the intervention and control arms during different time points

	6 months before the intervention Intervention N=140 Control N=104	0–6 months after the intervention Intervention N=135 Control N=153	6–12 months after the intervention Intervention N=96 Control N=77
Planned actions for risk for pressure ulcers, N (%)	51 (36.4) 46 (44.2)	56 (41.5) 52 (34.0)	39 (40.6) 26 (33.8)
Planned actions for *no* risk for pressure ulcers, N (%)	89 (63.6) 58 (55.8)	79 (58.5) 101 (66.0)	57 (59.4) 51 (66.2)
Planned actions for risk for malnutrition, N (%)	84 (60.0) 70 (67.3)	100 (74.1) 91 (59.5)	72 (75.0) 47 (61.0)
Planned actions for *no* risk for malnutrition, N (%)	56 (40.0) 34 (32.7)	35 (25.9) 62 (40.5)	24 (25.0) 30 (39.0)
Planned actions for risk for poor oral health, N (%)	40 (28.6) 56 (53.8)	54 (40) 82 (53.6)	46 (47.9) 43 (55.8)
Planned actions for *no* risk for poor oral health, N (%)	100 (71.4) 48 (46.0)	81 (60.0) 71 (46.4)	50 (52.1) 34 (44.2)
Planned actions for risk for falls, N (%)	121 (86.4) 85 (81.7)	118 (87.4) 123 (80.4)	79 (82.3) 67 (87.0)
Planned actions for *no* risk for falls, N (%)	19 (13.6) 19 (18.3)	17 (12.6) 30 (19.6)	17 (17.7) 10 (13.0)

### Implemented planned actions

Of the 299 older persons for whom actions were planned, implementation occurred for 270 (90.3%). Throughout the study period, a total of 570 actions were implemented, representing 80.9% of the planned actions.

In the intervention arm, 310 planned actions (54.4%) were implemented during the study period. Specifically, 140 planned actions (45.2%) were implemented 6 months before the intervention, 129 (41.6%) 0–6 months after the intervention and 41 (13.2%) 6–12 months after the intervention. In the control arm, 260 planned actions (46.6%) were implemented during the study period. Of these planned actions, 100 (38.5%) were implemented 6 months before the intervention, 135 (51.9%) 0–6 months after the intervention and 25 (9.6%) 6–12 months after the intervention. However, as shown in table 4, the intervention arm had more implemented actions for all the risks 0–6 months after the intervention, compared with the control arm. Additional information regarding the implementation of the planned actions for all risks in both study arms at different assessment points are provided in table 4. Table 4 also provides information on older persons whose planned actions were not implemented despite being at risk.

**Table 4:** Implemented planned actions (n=570) for older persons assessed for the risks of pressure ulcers, malnutrition, poor oral health and falls in the intervention and control arms during different time points

	6 months before the intervention Intervention N=140 Control N=100	0–6 months after the intervention Intervention N=129 Control N=135	6–12 months after the intervention Intervention N=41 Control N=25
Implemented planned actions of risk for pressure ulcers, N (%)	44 (31.4) 31 (29.8)	49 (36.3) 22 (14.4)	14 (42.7) 4 (5.2)
*Not* implemented planned actions of risk for pressure ulcers, N (%)	96 (68.6) 69 (66.3)	80 (59.6) 113 (73.9)	27 (28.1) 21 (77.3)
Implemented planned actions of risk for malnutrition, N (%)	71 (50.7) 50 (48.1)	90 (66.7) 46 (30.1)	28 (29.2) 10 (32.5)
*Not* implemented planned actions of risk for malnutrition, N (%)	69 (49.3) 50 (48.1)	39 (28.9) 89 (58.2)	13 (13.5) 15 (19.5)
Implemented planned actions for poor oral health, N (%)	33 (23.6) 36 (34.6)	49 (36.3) 39 (25.5)	16 (16.7) 12 (15.6)
*Not* implemented planned actions for poor oral health, N (%)	107 (76.4) 64 (61.5)	80 (59.3) 96 (62.7)	25 (26.0) 13 (16.9)
Implemented for planned actions for risk of fall, N (%)	100 (71.4) 59 (56.7)	105 (77.8) 65 (42.5)	31 (32.3) 19 (24.7)
*Not* implemented actions for risk of fall, N (%)	40 (28.6) 41 (39.4)	24 (17.8) 70 (45.8)	10 (10.4) 6 (7.8)

## Discussion

This section discusses the results of the recruitment, randomisation and retention of nursing homes as well as the clinical outcomes required for a definitive trial. These results are evaluated against the predefined progression criteria (table 1) to determine whether proceeding to a definitive trial is warranted.

Although the recruitment of nursing homes is widely recognised as time-consuming and challenging,^32^ our recruitment strategy proved successful (although demanding) according to the predefined criteria (ie, ≥8 nursing homes). The structured step-by-step recruitment strategy may explain our recruitment success. First, we engaged early and collaborated closely with heads of nursing homes in the municipality. This likely reflected and increased the perceived relevance of the study, as leadership engagement in nursing homes has proven to be crucial for successful implementation in similar settings.^33 34^ Second, the use of digital recruitment methods might have enabled the managers of eligible nursing homes to easily attend to informational meetings, thereby lowering logistical barriers and facilitating broader participation. Moreover, the nursing home managers who expressed interest in participating received additional information in both written and video format, which possibly enhanced their understanding and commitment. Providing adequate information appears particularly important in codesign projects, as unmet expectations were a key concern highlighted in our previous study.^35^ Moreover, consistent with results from other studies,^33 36^ the ongoing dialogue and engagement with both municipal and nursing home managers likely contributed to the strong interest in sustained involvement in the study, evidencing the predefined progression criteria regarding absolute retention rate (ie, ≥ 6 nursing homes). Nevertheless, despite the successful recruitment and retention achieved in this pilot study, it is important to acknowledge the potential methodological limitations related to these processes. First, participating nursing homes may have been particularly motivated to engage in the study, potentially introducing selection bias. Second, we observed that nursing homes involved in ongoing internal projects or those undergoing organisational changes such as the appointment of new managers, were more likely to decline participation. These insights highlight the importance of timing and organisational stability when recruiting nursing homes for the definitive trial. To address this, we plan to apply broad inclusion criteria that better reflect the diversity of real-world settings, by including those with variety levels of experience using Senior Alert and differing degrees of motivation to participate. This approach aims to capture a more representative picture of real-world conditions and practices, thereby informing the trial design and enhancing the generalisation of the findings.^37^

Given the nature of the intervention, blinding of allocation was not possible.^37^ Importantly, nursing homes were recruited prior to randomisation and only informed about allocation output afterwards.^38^ Regarding randomisation, using Microsoft Excel for this process was straightforward and technically unproblematic. A formula was applied to allocate nursing homes to either intervention or control arms, and the procedure proved logistically feasible according to the predefined progression criteria. This approach demonstrates both the simplicity and methodological soundness in randomising clusters using Microsoft Excel, and it might inspire future trials. However, the methodological limitations of the randomisation process must be acknowledged. This process led to baseline imbalance (ie, differences in nursing home type, the number of older persons living there and the number of nursing staff working there) between the intervention and control arms. Nevertheless, this was anticipated due to the small sample size, and since the current study was designed as an external pilot study, the primary outcome was to assess the randomisation procedure rather than to evaluate the effectiveness of the intervention.^25^ In the future definitive trial, an adequately powered trial^39^ and stratified randomisation (type and size of nursing homes) may reduce such imbalances and ensure better baseline comparation.^40^

To our knowledge, this is the first pilot study to evaluate the entire preventive care process targeting pressure ulcers, malnutrition, poor oral health and falls among older persons in nursing homes as outlined by Senior Alert, contributing to valuable knowledge in this field. Additionally, it is the first pilot study to evaluate the intervention. Our results showed that extracting and analysing data from Senior Alert was feasible, thus meeting the progression criteria regarding the intended clinical outcomes. Although the current study was not powered for hypothesis testing, several findings raise concern. Notably, more than 16% of the older persons in the recruited nursing homes were not risk assessed before the intervention started, leading to their exclusion from the study. This result is particularly relevant to Swedish nursing homes since the guidelines stipulate that all older persons should be risk assessed no later than 3 weeks after moving into a nursing home and should receive follow-up assessments at least every 6 months thereafter. Moreover, given the known high prevalence of the risks for pressure ulcers, malnutrition, poor oral health, and falls and considering that approximately 90% of older persons in our previous study^3^ present with at least one of these health risks, this gap in risk assessment is particularly worrying. Although our results emphasises the potential relevance of the current intervention in enhancing nursing home staff’s knowledge to risk prevention, our previous study identified several barriers to the use of the Senior Alert registry.^4^ The registration process was perceived as complex, time-consuming and of limited added value, contributing to a sense of double documentation. Importantly, staff also described an alternative approach in which general preventive care actions were implemented, rather than being strictly guided by individual risk assessments.^4^ This suggests that a more universal preventive approach may be both feasible and acceptable in this context. Such an approach should be considered in the definitive trial, as it may help mitigate reduce screening fatigue among staff.

Our results indicate that more care actions were planned to prevent pressure ulcers, malnutrition, and falls in the intervention arm than in the control arm 0–6 months after the intervention. Similarly, more care actions were implemented to prevent pressure ulcers, malnutrition, poor oral health, and falls in the intervention arm than in the control arm at the same assessment point. These results suggest that the intervention may positively influence the preventive care process as outlined by Senior Alert and, potentially, reduce the burden of these risks on older persons. However, these initial improvements could be associated with methodological limitations related to initial engagement among staff and should therefore be interpreted with caution. Notably, our results indicate a reduction in both planned and implemented actions for all risks in both study arms 6–12 months after the intervention. This reduction may reflect the burden of reported assessments and documentation over time. From a methodological perspective, this represents an important perspective when interpreting longitudinal trends in registry-based preventive care. One explanation could be that registering planned and implemented actions may simply be deprioritised or omitted. In fact, registries are known to be vulnerable to missing data. Such methodological limitations have also been noted in similar studies using a similar design.^41 42^ Another explanation could be that nursing home staff often find their work highly demanding,^4^ with the majority reporting symptoms of burnout.^43^ Additionally, the observed reduction may be related to staff turnover, leading to a gradual loss of intuitional knowledge, rather than solely reduced engagement over time. This result has important implications for the design of the definitive trial. Rather than relying primarily on single replenishment, such as a booster session at 6 months, we should explicitly account for the high levels of staff turnover in nursing homes.^44^ One strategy is continuous onboarding, whereby the intervention is systematically integrated into the organisational routines and initial training of all newly employed staff. Integrating the intervention within standard introduction processes may help maintain continuity and prevent the loss of institutional knowledge associated with staff turnover. Yet, findings from our previous study indicate that onboarding strategies alone are insufficient.^35^ Nursing home staff emphasised the importance of sustained organisational and managerial support to effectively carry out preventive work, pointing to leadership as a critical enabling factor. Therefore, the intervention should also include structured local working routines that clearly incorporate leadership engagement and support. This aligns with prior research in similar settings, demonstrating that strong and supportive leadership is essential for successful implementation of interventions.^45^ Consequently, the observed reduction in planned and implemented actions may be attributed to a combination of actual behavioural change and incomplete registrations, underscoring the need for qualitative follow-ups to better understand the mechanism behind this pattern.

Although the descriptive trends do not indicate a sustained increase in care actions over time, it is important to emphasise that the primary aim of the present pilot study was not to evaluate efficacy,^25^ but to determine whether progression to a definitive trial is warranted based on predefined progression criteria, as recommended by the literature.^24^ From this perspective, the lack of a stable plateau, alongside the initial increase in care actions observed in the intervention arm, should be interpreted as informative. Indeed, the observed trend suggests that while the intervention may initially stimulate engagement, it is not yet sufficiently embedded within routine practice to sustain change over time. This raises important considerations regarding its fit within the high-pressure environment of nursing homes. Despite the close collaboration with stakeholders during the development and feasibility testing phases, the intervention in its current form appears to be insufficiently integrated into existing workflows to support long-term maintenance. This should not be interpreted solely, rather, consistent with the MRC framework for developing and evaluating complex interventions,^17^ these results highlight the need for further refinement of the intervention itself prior to the next phase. Importantly, when comprehensively evaluated against the predefined progression criteria, including the success of the recruitment, randomisation and retention of nursing homes as well as the successful extraction of the clinical outcomes, the results of the current study support progression to a definitive trial. However, such trial should incorporate embedded qualitative research to distinguish between failures in the provision of preventive care or limitations in registration practices within Senior Alert.

## Conclusions

Conducting this external pilot study was a crucial step in deciding whether to proceed with the definitive trial. The progression criteria in our study served as guidelines rather than strict rules. Based on a comprehensive evaluation of these criteria, our codesigned intervention—which aimed at preventing pressure ulcers, malnutrition, poor oral health and falls among older persons in nursing homes—sufficiently justifies a future definitive trial. Furthermore, since pilot studies in nursing remain underreported,^46^ the current study findings add valuable insights to the field.

## Data Availability

No data are available.
