# Supplementary material for: Codesigned STAIR OF KNOWLEDGE intervention to prevent pressure ulcers, malnutrition, poor oral health and falls in Swedish nursing homes: an external pilot cluster randomised controlled study
Source: BMJ Open. 2026 Jun 25;16(6):e118018. doi: 10.1136/bmjopen-2026-118018 (PMC13311616; doi:10.1136/bmjopen-2026-118018)
Supplement: Supplementary file 1 [file bmjopen-2026-118018.reviewer_comments.pdf]

PEER REVIEW HISTORY

BMJ Open publishes all reviews undertaken for accepted manuscripts. Reviewers are asked to complete a checklist review form and are provided with free text boxes to elaborate on their assessment. These free text comments are reproduced below.

ARTICLE DETAILS

Title (Provisional)

The codesigned STAIR OF KNOWLEDGE intervention to prevent pressure ulcers, malnutrition, poor oral health, and falls in Swedish nursing homes: An external pilot cluster randomized controlled study

Authors

Neziraj, Merita; Kumlien, Christine; Axelsson, Malin

VERSION 1 - REVIEW

**Reviewer**	**1**
**Name**	**Falandry, Claire**
**Affiliation**	**Hospices Civils de Lyon**
**Date**	**29-Mar-2026**
**COI**	**None**

- General Overview

The manuscript describes an external pilot cluster randomized controlled trial (RCT) evaluating the feasibility of the "STAIR OF KNOWLEDGE" intervention. The study is grounded in the MRC and Knowledge to Action (KTA) frameworks and addresses a critical public health issue: the evidence-practice gap in nursing home preventive care. While the study successfully meets its predefined technical progression criteria (recruitment and retention), the descriptive data reveal significant "red flags" regarding the intervention’s long-term sustainability and clinical impact that must be addressed before a definitive trial is launched.

- Major Revision Points

A. Long-term Sustainability and the "Staff Dilution" Effect

The data in Table 4 show a concerning collapse in implemented actions between 6 and 12 months in the intervention group (dropping from 129 to 41 actions).

The authors attribute this to "burnout," but this fails to account for the high staff turnover typical of nursing homes. In these settings, an intervention "suffocates" not just because of exhaustion, but because trained staff leave and are replaced by uninitiated personnel. This leads to a rapid dilution of the intervention’s "dosage" within the team.

=> The authors must discuss a strategy for the definitive trial that goes beyond a simple "booster session." They should explore a model of continuous onboarding, where the intervention is integrated into the orientation of every new hire to prevent the loss of institutional knowledge.

B. Questionable Clinical Trends and "Novelty Bias"

Although such a pilot is not powered for efficacy, the descriptive trends are not favorable. The slight increase in actions at 0–6 months in the intervention arm likely reflects a Hawthorne effect (novelty bias) rather than a structural change in care culture.

=> If the intervention cannot maintain a stable plateau of care even in a small, highly monitored pilot group, the authors must justify the utility of a larger, more expensive definitive trial. They should discuss whether the intervention, in its current complex form, is truly adapted to the high-pressure environment of a modern nursing home.

C. Universal Precaution vs. Individual Screening Efficiency

The study reports a risk prevalence of >80% for falls and malnutrition. Furthermore, 16% of residents were excluded simply because staff failed to complete the initial screening.

=> When a condition is nearly universal, individualized screening becomes a bureaucratic bottleneck rather than a clinical aid. The authors should discuss the potential for a "Universal Precaution" approach, where core preventive actions (e.g., hydration protocols, oral hygiene standards) are implemented by default for the entire population. This would reduce "screening fatigue" and the 16% exclusion bias identified in this pilot.

D. Selection Bias and Generalizability

Only 8 out of 21 eligible nursing homes (38%) participated. Establishments undergoing management changes or internal crises explicitly declined.

=> The "feasibility" proven here applies only to a subset of highly motivated, stable facilities. The authors need to address how they will mitigate this selection bias in a definitive trial to ensure the intervention works in "average" or "high-stress" facilities, not just the "best-case" ones.

- Structural and Minor Points

1. Visual Presentation of the Trial (CONSORT Flow Chart)

The trial flow is currently described in dense prose, making it difficult to track the movement of clusters and individuals.

=>: The authors should provide a formal CONSORT Flow Diagram for randomized pilot and feasibility trials. This should clearly visualize the attrition from the initial 39 facilities down to the final analyzed residents, including the 16% excluded due to missing baseline data.

2. Baseline Imbalance

Randomization resulted in a significantly larger control arm (n=184 vs. n=118).

=> While the authors mention stratified randomization for the future, they should specifically identify which variables (e.g., facility size, specialized dementia vs. general care units) they recommend for stratification to ensure better balance in the definitive trial.

3. Data Integrity and Documentation

There is a high risk that care is being provided but not "clicked" in the Senior Alert registry due to time constraints.

=> The authors should discuss using "data triangulation" (e.g., qualitative interviews or spot audits) in the future trial to distinguish between a failure to provide care and a failure to document care.

=> Recommendation: Major Revision.

While the trial is technically feasible, the intervention itself shows signs of "futility" at the 12-month mark. The authors should address in their discussion the structural issues of staff turnover and screening efficiency before it can be concluded that the protocol can be considered ready for a large-scale definitive trial.

**Reviewer**	**2**
**Name**	**Holloway, Samantha**
**Affiliation**	**Cardiff University**
**Date**	**31-Mar-2026**
**COI**	**None**

This is an interesting study on an important topic. Overall the pilot study is reported well. There are just some minor edits required to improve the reporting:

- The results section in the Abstract could provide more detail

- Specific inclusion (and exclusion) criteria seem to be missing

Please see the attached annotated PDF.

For the future it would be interesting to include not only Pressure Ulcers, but also other conditions such as Moisture-Associated Skin Damage and Skin Tears as there are shared risk factors for all these. This is not a requirement for this manuscript just a suggestion for further research.

- Additional Figures to summarise the steps in the process would be helpful

**** The reviewer provided a marked copy with additional comments. Please contact the publisher for full details. ****

VERSION 1 - AUTHOR RESPONSE

Reviewer: 1
Dr. Claire Falandry, Hospices Civils de Lyon, University of Lyon
Comments to the Author:
- General Overview

The manuscript describes an external pilot cluster randomized controlled trial (RCT) evaluating the feasibility of the "STAIR OF KNOWLEDGE" intervention. The study is grounded in the MRC and Knowledge to Action (KTA) frameworks and addresses a critical public health issue: the evidence-practice gap in nursing home preventive care. While the study successfully meets its predefined technical progression criteria (recruitment and retention), the descriptive data reveal significant "red flags" regarding the intervention's long-term sustainability and clinical impact that must be addressed before a definitive trial is launched.

Authors replay: Thank you for your valuable feedback. We have revised the manuscript in line with all your comments, please see below. Your feedback helped to improve our manuscript.

- Major Revision Points

A. Long-term Sustainability and the "Staff Dilution" Effect
The data in Table 4 show a concerning collapse in implemented actions between 6 and 12 months in the intervention group (dropping from 129 to 41 actions).
The authors attribute this to "burnout," but this fails to account for the high staff turnover typical of nursing homes. In these settings, an intervention "suffocates" not just because of exhaustion, but because trained staff leave and are replaced by uninitiated personnel. This leads to a rapid dilution of the intervention's "dosage" within the team.
=> The authors must discuss a strategy for the definitive trial that goes beyond a simple "booster session." They should explore a model of continuous onboarding, where the intervention is integrated into the orientation of every new hire to prevent the loss of institutional knowledge.

Author reply: We appreciate your comments and fully agree with you. In response to these comments, the discussion has been revised to further elaborate on the intervention's long-term sustainability addressing staff turnover and strategies to maintain sustainability. Please see grey-coloured text on pages 19 and 20.

B. Questionable Clinical Trends and "Novelty Bias"
Although such a pilot is not powered for efficacy, the descriptive trends are not favorable. The slight increase in actions at 0–6 months in the intervention arm likely reflects a Hawthorne effect (novelty bias) rather than a structural change in care culture.
=> If the intervention cannot maintain a stable plateau of care even in a small, highly monitored pilot group, the authors must justify the utility of a larger, more expensive definitive trial. They should discuss whether the intervention, in its current complex form, is truly adapted to the high-pressure environment of a modern nursing home.

Author reply: Thank you for highlighting this important matter. We agree with you that the current pilot study was not powered for efficacy, which we in the revised manuscript discuss in relation to potential novelty bias, which not necessarily preclude a real increase. Please see yellow-colorized text on pages 20 and 21.

C. Universal Precaution vs. Individual Screening Efficiency
The study reports a risk prevalence of >80% for falls and malnutrition. Furthermore, 16% of residents were excluded simply because staff failed to complete the initial screening.
=> When a condition is nearly universal, individualized screening becomes a bureaucratic bottleneck rather than a clinical aid. The authors should discuss the potential for a "Universal Precaution" approach, where core preventive actions (e.g., hydration protocols, oral hygiene standards) are implemented by default for the entire population. This would reduce "screening fatigue" and the 16% exclusion bias identified in this pilot.

Author reply: Thank you forraising an important point, which we address in the revised manuscript by discussing individualized screening versus universal precaution approach. Please see red-colorized text on page 19.

D. Selection Bias and Generalizability
Only 8 out of 21 eligible nursing homes (38%) participated. Establishments undergoing management changes or internal crises explicitly declined.
=> The "feasibility" proven here applies only to a subset of highly motivated, stable facilities. The authors need to address how they will mitigate this selection bias in a definitive trial to ensure the intervention works in "average" or "high-stress" facilities, not just the "best-case" ones.

Author reply: Thank you for bringing this to our attention. We have revised the discussion to address selection bias and generalizability. Please see green-colorized text on page 17.

- Structural and Minor Points

1. Visual Presentation of the Trial (CONSORT Flow Chart)
The trial flow is currently described in dense prose, making it difficult to track the movement of clusters and individuals.
=>: The authors should provide a formal CONSORT Flow Diagram for randomized pilot and feasibility trials. This should clearly visualize the attrition from the initial 39 facilities down to the final analyzed residents, including the 16% excluded due to missing baseline data.

Author reply: In line with your suggestion, we have added a CONSORT Flow Diagram visualizing the attrition process. Please see Figure 1. Also added in page 9, see green-colorized text.

2. Baseline Imbalance
Randomization resulted in a significantly larger control arm (n=184 vs. n=118).
=> While the authors mention stratified randomization for the future, they should specifically identify which variables (e.g., facility size, specialized dementia vs. general care units) they recommend for stratification to ensure better balance in the definitive trial.

Author reply: Good point, we have clarified that type and size of nursing homes are recommended to take into consideration in future stratified randomization to ensure better balance in a definitive trial. Please see purple-colorized text on page 18.

3. Data Integrity and Documentation
There is a high risk that care is being provided but not "clicked" in the Senior Alert registry due to time constraints.
=> The authors should discuss using "data triangulation" (e.g., qualitative interviews or spot audits) in the future trial to distinguish between a failure to provide care and a failure to document care.

Author reply: We agree with you and have extended the discussion to include recommendation about data triangulation in terms of qualitative research method in the future trial. Please see green-colorized text on page 21.

=> Recommendation: Major Revision.
While the trial is technically feasible, the intervention itself shows signs of "futility" at the 12-month mark. The authors should address in their discussion the structural issues of staff turnover and screening efficiency before it can be concluded that the

protocol can be considered ready for a large-scale definitive trial.

Author reply: We appreciate your thoughtful comments, which have been addressed in an expanded discussion.

Reviewer: 2
Mrs. Samantha Holloway, Cardiff University
Comments to the Author:
This is an interesting study on an important topic. Overall the pilot study is reported well. There are just some minor edits required to improve the reporting:
- The results section in the Abstract could provide more detail
- Specific inclusion (and exclusion) criteria seem to be missing

Author replay: Thank you for your positive feedback. We have revised the results in the abstract and added inclusion criteria in the paragraph “Clinical outcomes: Data extraction and statistical analysis” in accordance with your comments. Please see yellow text in on pages 2 and 8**.**

Please see the attached annotated PDF.

Author replay: Thank you for your comments**.**

- Thank you for noticing. However, the bullet points in the section Strengths and limitations of this study on page 3 are revised in accordance with the editor’s requirements.
- We clarified the stakeholders in the paragraph “Patient and public involvement”, marked with yellow on page 7.

For the future it would be interesting to include not only Pressure Ulcers, but also other conditions such as Moisture-Associated Skin Damage and Skin Tears as there are shared risk factors for all these. This is not a requirement for this manuscript just a suggestion for further research.

Author replay: Thank you for your important suggestion, which is worth considering in our future research.

- Additional Figures to summarise the steps in the process would be helpful

Author replay: We have added CONSORT Flow Diagram to illustrate the process, in line with your suggestion. Please see Figure 1. Also added in page 9, see green-colorized text.

Reviewer: 1

If you have selected ‘Yes’ above, please provide details of any competing interests.: Not applicable

Reviewer: 2
If you have selected ‘Yes’ above, please provide details of any competing interests.: Not applicable
